# The views and experiences of people with myeloma referred for autologous stem cell transplantation, who declined to participate in a physiotherapist-led exercise trial: a qualitative study

**DOI:** 10.1080/09593985.2023.2244068

**Published:** 2023-08-09

**Authors:** Orla McCourt, Abigail Fisher, Joanne Land, Gita Ramdharry, Kwee Yong

**Affiliations:** a Therapies and Rehabilitation, University College London Hospitals NHS Foundation Trust, London, UK; b Research Department of Haematology, UCL Cancer Institute, University College London, London, UK; c UCL Department of Behavioural Science and Health, University College London, London, UK; d Queens Square Centre for Neuromuscular Diseases, National Hospital for Neurology and Neurosurgery, UCLH NHS Trust/UCL Institute of Neurology, University College London, London, UK

**Keywords:** Myeloma, cancer rehabilitation, physiotherapy, exercise, qualitative research, recruitment, research participation

## Abstract

**Background:**

Recruitment rates to rehabilitation trials are variable among cancer survivors, and deeper investigation into the causes for declining participation is needed. The aim of this study was to qualitatively explore the experiences of people with myeloma referred for autologous stem cell transplant who were approached to take part in a physiotherapist-led exercise trial but declined.

**Methods:**

Participants were asked to participate in this qualitative study after declining to participate in a trial conducted at a UK tertiary cancer center. Semi-structured interviews were conducted. Data was analyzed inductively using reflexive thematic analysis.

**Results:**

Interviews from 18 myeloma patients (56% male, mean age 62 years) were analyzed. Four themes were identified: 1) Traveling to the specialist center is challenging, not just logistically; 2) Individualized approach valued but recall of research information variable; 3) Being less active has profound impact yet ameliorative support is lacking; and 4) Common side-effects of treatment are expected and endured but personal impact underestimated and unaddressed.

**Conclusion:**

A number of barriers to participation were identified. Travel, a commonly cited reason for declining research participation, is more than a logistical issue for cancer survivors experiencing side-effects and the time burden of clinical appointments. Expectation or knowledge of the typical side-effects from myeloma and its treatment may lead to under-reporting of concerns to care providers, despite their impact upon daily activities and quality of life. Approaches used for research recruitment should consider the timing and consequences of ongoing cancer treatment to reduce potential barriers to participation.

## Introduction

Myeloma is a hematological malignancy, which affects the plasma cells in the bone marrow (Brown, 2017). Myeloma is a relapsing-remitting cancer: periods of active, symptomatic disease that require intensive treatment, are separated by periods of stable disease or plateau phases where no, or only maintenance treatment, is required. Incidence of myeloma increases with age, with a marked increase in incidence from 55 years and affects approximately 18 per 100,000 people in the UK (Haematological Malignancy Research Network, 2019). Although incurable, improved understanding of the disease mechanisms along with advances in treatments over the last decade has meant that survival in myeloma is increasing at the fastest rate among all cancer types in the UK (Myeloma, 2018) and contributes most to the increasing survival rates of hematological cancers generally (Blood Cancer UK, 2019).

More physically active myeloma survivors have improved quality of life (QOL), reduced treatment-related side-effects and lower fatigue compared to those who are less active (Jones et al., 2004; Servadio et al., 2020), indicating a role for supporting maintenance or improvement of physical activity (PA) in this population. Exercise trials in myeloma have been unable to definitively determine efficacy in this population due to heterogeneous and underpowered trial designs. They did, however, demonstrate that exercise is safe with some evidence for positive effects on physical, psychological and QOL outcomes (Gan, Sim, and Santorelli, 2016; Smith et al., 2015).

Randomized controlled trials (RCTs) are often considered a “gold standard” experimental research design (Begg et al., 1996) and are commonly used to evaluate healthcare interventions, yet up to 50% do not successfully recruit their target sample size without extension of funding and/or time (Houghton et al., 2020). Challenges with recruitment can compromise validity of RCTs through selection bias, underrepresentation of participants with particular characteristics, socio-economic background or those with particular interest in the intervention being trialed. RCTs of behavioral interventions, such as exercise interventions, are considered particularly challenging to recruit to with estimated uptake differing depending on recruitment strategies and research setting (Foster et al., 2011). A meta-analysis of recruitment and retention of exercise RCTs in people with multimorbidity found the pooled recruitment rate of 21 RCTs to be 74% (Harris et al., 2021). Specifically, in exercise oncology, recruitment rates have been reported to be between 10% and 44% in solid tumor patient groups (Strandberg et al., 2022) with higher rates reported in those with advanced cancer diagnoses (Sheill et al., 2019). Recruitment rates of 50% have been reported in exercise trials delivered during stem cell transplant, but reasons for declining participation were not detailed (Wood et al., 2016). A previous myeloma survivorship exercise RCT at our center, which used an adapted Zelen design, had an uptake of 57% (Koutoukidis et al., 2020; Land et al., 2020). However, the single-arm pilot study preceding that RCT recruited 80% of approached patients (Groeneveldt et al., 2013). The lower uptake among participants randomized to the intervention arm of the RCT was explained by participants citing the extra time or travel commitment involved in taking part in the intervention but the reason for such a difference in uptake between the pilot and RCT is not clear. Variations in uptake across trials are possibly related to heterogeneity of study design, recruitment strategies and follow-up procedures, but this variation presents uncertainty when planning future exercise oncology RCTs, even those with successful pilots.

Reporting guidelines for research studies, such as the Consolidated Standards of Reporting Trials (CONSORT) guidance (Schulz, Altman, Moher, and CONSORT Group, 2010), advise provision of reasons for potential participants declining or being ineligible when reporting enrollment of an RCT. This information allows assessment of the representativeness (National Institute for Health Research, 2020) and external validity of study (Moher et al., 2012) as well as the acceptability of the intervention. However, reasons for declining to take part are often provided as brief descriptions without context and do not contribute to understanding cancer survivors’ decision-making in relation to acceptability of the trial design or intervention on offer. Using pilot studies to estimate recruitment rate is important part of establishing feasibility for a larger trial, but it is also vital for evaluation of any potential volunteer bias. A number of studies have reported deeper exploration of reasons for nonparticipation in trials (Attwood et al., 2016; Featherstone and Donovan, 2002). Influences on participation in RCTs have been explored in numerous qualitative studies (Houghton et al., 2020) although deeper understanding of why people with cancer may decide not to take part in exercise-related research is required. Findings could further facilitate improved study and intervention design to attract participants who may be considered harder to recruit. The aim of this study was to explore the experiences of people with myeloma referred to a specialist cancer center for consideration for autologous stem cell transplant (ASCT), who were approached to take part take part in an exercise-related research trial but declined. Their experiences of discussing PA with healthcare team since diagnosis was also investigated.

## Methods

### Design

This qualitative study was embedded within the recruitment process of the PERCEPT myeloma study, a pilot RCT of an exercise prehabilitation and rehabilitation exercise intervention delivered as part of the ASCT pathway (McCourt et al., 2020; McCourt et al., 2023) which was pre-registered (ISRCTN15875290). Guidelines for reporting qualitative research were followed (O’Brien et al., 2014).

### Participants

Participants were people living with myeloma referred to a specialist cancer center in the city of London, United Kingdom, for an ASCT following induction treatment at local hospitals. They were deemed eligible to take part in the PERCEPT RCT, had been contacted by telephone to introduce the trial and sent a participant information sheet (PIS) but ultimately declined to enroll. After an initial approach, participants were given time to consider and confirm that they did not wish to take part in the trial. They were asked if they would be willing to participate in a qualitative interview discussing their reasons for declining. Written informed consent was obtained prior to interviews. Ethical approval to interview decliners was obtained as part of approval for the PERCEPT myeloma study (London – Camden and Kings Cross Research Ethics Committee reference 19/LO/0204).

### Qualitative interviews

Semi-structured interviews were used to gather data. An interview schedule was developed by the lead author to address the aims of the study and guide the interviews (Supplementary File). The lead author, a female clinical academic physiotherapist, was the chief investigator and sole recruiter for the trial and conducted all the interviews. The interviews were conducted face-to-face or by telephone depending on participant preference, were audio recorded, fully anonymized and transcribed verbatim by a professional transcription service. Recruitment ceased when no additional data related to the research questions were arising from the interviews and sufficient data were obtained to allow patterns to be identified across the data (Saunders et al., 2018). Participants were not offered any incentives for participation.

### Analysis

Demographics of the participants were collected from medical notes. In order to provide context for understanding the geographical challenges explored in the study, distances between each participant’s home and the specialist cancer center were calculated by shortest estimated land route and in relation to the Greater London area using an online mapping tool (https://www.freemaptools.com/distance-between-uk-postcodes.htm).

Reflexive thematic analysis (TA) (Braun and Clarke, 2006, 2013) was chosen for the qualitative analysis. It incorporates procedures and underlying research values that are fully qualitative (Braun and Clarke, 2020) and is considered a robust method that can result in nuanced, interpretive and complex analysis (Braun, Clarke, and Weate, 2016). Alongside reflexivity, TA also requires deliberate and considered choice of ontological and epistemological frameworks that underpin the research methods and findings generated. An ontological orientation of realism (i.e. belief that the study and the process of being approached to take part given a PIS and followed up in clinic to consent, exist in a real-world way independently of any perceptions or constructions) with an epistemological orientation of constructivism that peoples’ understanding of the study, the approach and their reasons for not taking part are shaped by their prior experiences and assumptions underpinned this analysis. Therefore, an inductive approach underpinned by critical realist onto-epistemology was used (Mukumbang, 2023).

The lead author led the reflexive TA process, an iterative process involving six phases (Braun and Clarke, 2013). Firstly, interviews were listened to repeatedly for familiarization with the content and to check the transcripts for accuracy. Transcripts were then analyzed through repeated reading and assignment of coding labels to the text. A second researcher double-coded two (11%) of the transcripts. Memos and notes were recorded throughout the coding phase which informed and confirmed ideas generated through engaging with the data. Repeated rounds of analysis were carried out iteratively revising and discarding codes and sets until the themes and subthemes were developed and described. Data analysis was conducted using NVivo 12.

## Results

Between June 2019 and January 2020 58 people were approached for the PERCEPT trial. Of these, 29 (50%) consented to take part and 29 declined. All of those who declined were approached for the current study. Of these, 23/29 (79%) completed a consent form and provided an e-mail address and telephone number. Ten percent (3/29) participants could not be contacted to arrange an interview and 1 (4%) withdrew consent and declined an interview. Eighty percent (19/23) participants took part in interviews. Data collection was ceased at 19 interviews as no new views relevant to the main research questions were arising from interviews (Saunders et al., 2018). One interview was not recorded in error, therefore 18 were available for analysis. Interview time ranged from 14 to 37 minutes (mean 24 minutes). Sixteen interviews were conducted over the telephone. Of the two interviews conducted face-to-face, one participant had their wife present and the other had their son present.

The sample of 18 participants interviewed ranged in age from 41 to 73 years (mean age 62 years [SD 8]) and 10 (56%) were male. Participants were median 7 months post-diagnosis (range 3–80 months). Participants lived on average 38 miles (61.2 km) by shortest land transport route (SD 22, range 5–73 miles) from the specialist cancer center. The majority of participants (13/18, 72%) lived outside of the Greater London region (Table 1).

**Table 1.** Interviewee characteristics.

	Pseudonym	Age at interview	Sex	Time since diagnosis (months)	Living within Greater London
1	Ben	72	Male	8	No
2	Jim	69	Male	9	No
3	Ian	66	Male	3	No
4	Ann	69	Female	8	No
5	Lara	66	Female	6	No
6	Pete	58	Male	6	No
7	Tim	55	Male	5	Yes
8	Dave	68	Male	5	No
9	Tom	64	Male	7	No
10	Mary	62	Female	3	No
11	Sally	41	Female	8	No
12	Liz	63	Female	6	Yes
13	Greg	65	Male	6	No
14	Bob	55	Male	9	No
15	Jane	58	Female	4	No
16	Andy	73	Male	7	Yes
17	Sarah	49	Female	80	Yes
18	Pam	60	Female	11	Yes

Four main themes and nine related subthemes were identified: 1) Traveling to the specialist center is challenging, not just logistically; 2) Individualized approach valued but recall of research information variable; 3) Being less active has profound impact yet ameliorative support is lacking; and 4) Common side-effects of treatment are expected and endured but personal impact underestimated and unaddressed.

### Travelling to specialist center is challenging, not just logistically

The most common reason for declining to take part in the PERCEPT study was because of travel. Overwhelmingly, interviewees shared that traveling to the center was a decisive factor in declining to take part in the main study. Many highlighted the idea of having to attend the center more frequently, the time commitment of these extra visits, as well as the journey required as particular challenges. It was difficult to tease out which of these were most problematic and the following subthemes were conceptualized to describe the challenges perceived by interviewees when considering the possible travel required to take part in the study.

#### Not wanting to travel to specialist center more than necessary

Many expressed that they lived “far” from the center and described complex, multi-modal routes involving public and private transport and taking substantial amount of time. For most interviewees, traveling into the specialist center was only experienced once prior to interview as they were only recently referred for ASCT. There was a sense from the data that many participants anticipated that the addition of ASCT-related appointments to their continuing appointments at their local hospitals would be too much.

The thing is, it’s so consuming, coming up here and what you have to do and the travelling and the amount of appointments, plus we were fitting [local hospital] in with it as well, we had to go to [local hospital] as well for the two corresponding times. To be honest it was a nightmare, to try and arrange and get that in your head … But we’ve come here today and it wasn’t too bad. We’ve still got to come another four or five times or maybe more, but put that on top of the others as well, it’s a lot. Dave, 68

There was an acknowledgment by some that alignment of study visits with routine appointment visits was a welcome consideration, but weekly travel was not feasible. Some suggested less frequent visits, such as monthly, would be acceptable. Anticipation of frequent appointments at the specialist center was also compounded by interviewees reporting the time commitment of traveling and attending appointments there. Alongside detailed descriptions of their journeys and the time taken, there was also a sense that waiting around for appointments as much as the appointments themselves took a significant amount of time. Most interviewees felt a visit to the specialist center took the best part of the whole day, and they presumed that visits for the study gym attendance would result in a similar commitment, which was central to their decision not to take part.

From a design viewpoint, the PERCEPT study was focussed in part on the idea of utilizing the routine treatment free period between induction chemotherapy and ASCT as potentially an ideal time to introduce an exercise intervention in preparation for the next phase of treatment. However, it was underappreciated that this treatment free period would also be considered by participants as an opportunity to take time away from recurrent hospital visits and therefore an intervention based at the specialist center may not be welcomed by people at this stage.

We had just gone through six months of chemo, going into hospital every week, once a week, I’ve now got an opportunity for a month off from hospitals before the stem cell treatment starts again, and I need that time really to live life a bit. So the thought of going into hospital once a week for the next six to eight weeks is not really in line with what I want to do at the moment. Pete, 58

#### Journey is a physical challenge

Fatigue is the most common side-effect of treatment experienced by people with myeloma, and unsurprisingly fatigue was a key feature across the dataset. There were explicit references by most interviewees to how tiring traveling to the center is as well as how physical demanding some considered the journey. Some highlighted how aspects of travel were difficult or not possible due to physical concerns related to impairments from diagnosis or side-effects of induction chemotherapy.

I’m much more mobile now than what I was, but it’s still quite a physical challenge for me to get around for any length of time – walking along long connecting tube tunnels, for example, or dealing with loads of stairs and things like that, I can do it but it’s very very tiring. Lara, 66

Some mention overt references to not being “fit” enough to undertake aspects of the journey by public transport or not wishing to travel unaccompanied for fear of being pushed in moving crowds or worries about having to undertake prolonged standing on trains.

I think essentially if I was fit enough to travel backwards and forwards, I think it would benefit me no doubt, but unfortunately, because I’m not in that state at the moment, it’s not convenient for me. Andy, 73

And rush hour and the rest of it, I think I’m going to be pushed about a bit. I had my son with me so that was a shield, if you like, and he’s keeping an eye on me, but if I’m coming up here on my own that would be an issue, certainly on the train journeys and underground. Ben, 72

There was a sense from many that being accompanied to travel for study visits would be a necessity, in a similar nature to how they considered it important to have their partner or family member with them for medical appointments. For some, this was related to concern from their loved one regarding their safety in traveling alone, with one referring to a fear of collapsing on the train making their partner “dead against” his participation. For others, there was a sense of low confidence around traveling to the specialist center alone.

#### Anticipation of further side-effects influence preference to avoid travel

All the interviewees had been approached to take part in the PERCEPT study in the week leading up to their first appointment with the ASCT team, and for many it was the first time they had been counseled on what the process would involve, including additional tests prior to the stem cell harvesting, admission for transplant and the possible side-effects to expect. There was a sense from the data that interviewees were not only cognizant of the side-effects of treatment that they had already experienced but now aware of further possible consequences of their future treatment. Weighing up the possibility of these additional or possibly amplified side-effects appeared to influence some people’s weariness of additional travel.

The only thing that did put me off was that I didn’t know how I’d be on the treatment that I was going to receive, the stem cell treatment. That was the only thing that put me off, not knowing how it would be, because I’ve been briefed about all the things that could happen to me. John, 72

There was an indication that some interviewees were anticipating side-effects to occur earlier in the process than to be expected, for example, some referred to not knowing how they would feel after ASCT but were relating those uncertain feelings to their reluctance to travel in the period prior to transplantation.

### Individualized approach valued but recall of research information variable

Participants described that the point in their treatment journey when they were approached (on referral for consideration for ASCT) that “information overload” made the additional task of considering taking part in a research study or exercise intervention too difficult to fully consider. There was unanimous acceptance by interviewees to being approached about a study of this kind at this point in the pathway. So receiving additional engagement about the research study was not considered burdensome or unwelcome but it is possible that participation in the study did not receive the interviewees’ full consideration due to the amount of other information they report having to take on at this time.

Many expressed sentiments of gratitude for being considered to approach with some even sharing expressions of feeling wanted or sought out. This was coupled with a sense of regret by most of not being able to take part in the study, both for loss of potential personal gain and in an altruistic sense of contributing to work that may help others with myeloma.

I’m sorry that I couldn’t participate or didn’t want to participate in it because it sounded very interesting and anything that can help other people in a similar position I’m encouraged to do, but it was just logistics-wise it was not good for me. Greg, 65

This combination of altruism and regret at not participating in the exercise study may in reality have been the influencing factors for most wanting to take part in this qualitative study, with the sentiment of “if it will help others” a feature across the dataset.

#### Personalized discussion, not just a leaflet

There was a clear pattern of engagement described by interviewees that gave insights into how taking part in the PERCEPT study was considered. It was clear that being contacted individually by phone call prior to their ASCT clinic appointment and followed up in clinic a few days later rather than just receiving written information was appreciated by all those who were interviewed with many expressing gratitude for time taken to be sought out, spoken with and followed up in person in clinic.

I think the personal approach … It’s such a complicated thing, isn’t it? I think it’s hard to get things over just on paper … I was quite happy to listen to what you said, and I could ask questions, and you could explain in more detail. I think the personal approach is nice. It’s a good way. Ann, 69

You took the time to come and see me. It could have just been a piece of paper, but you contacted me and you put a lot of effort in, and came to see me, and tried to explain it to me. And obviously that pleased me as well. Ian, 66

There was a general opinion by participants that they received all the information they required to make their decision from the discussion in the phone call, introducing the study. Many expressed the approach allowed them to come to their decision without feeling pressure. Overall, there was a sense that despite also receiving written information, most participants had made their decision not to take part based on the discussion and information received during the phone call to introduce the research study.

#### Participant information sheet less influential than verbal information when deciding to participate

There was much variation between interviewees regarding recall of the research study details and interaction with the PIS provided after initial approach by phone call. Nearly all reported they did not read the PIS fully or some did not read it at all. Interviewees expressed that the phone call had provided sufficient information for them to make their decision and that was why they did not read the PIS fully.

I think a phone call was exactly the right approach. Because actually, although I didn’t continue to read on all the paperwork, and what have you, I didn’t feel I needed to in order to make my decision. Lara, 66

Others felt they were overwhelmed with information or lack of time to engage further with the written information.

It has been quite overwhelming and all the things that I’m going to be going through. Do you know what I mean? My mind is boggling from that without reading something extra at this moment. Pam, 60

Many interviewees discussed their decision-making in the context of assumed allocation to the exercise arm. Specific questioning and probing about details related to the study revealed mixed recall and inaccuracies about the nature of the research, particularly in relation to awareness of randomization or allocation to group. The inaccuracies of recall of study details, as well as confirmation of many that they had not engaged with the written information, indicated that for many the phone call discussion may have hindered further consideration of study information from the PIS.

### Being less active has profound impact yet ameliorative support is lacking

The impact of initial diagnosis and/or subsequent induction chemotherapy treatment on levels of PA or function was highlighted by nearly all the interviewees, with fatigue, low energy or low stamina described as the main precipitating factor. Daily activities and ability to walk or run as a form of exercise were described by some interviewees as more challenging.

I can walk for an hour, but nowhere near the distance or speed that I could do before. Gardening is much more difficult now than it was before. So my mobility is certainly affected by the myeloma. Pete, 58

A number described experiencing the cycle of fatigue, resulting in less activity, leading to less capability carry out certain activities, although many did not recognize this debilitating cycle. There was a sense of fear in not knowing how fatigue might affect their ability to carry out an activity, especially outside of the home, so these activities were avoided.

I do try and move about but if I move about for about five, ten minutes, I have to sit down. It’s like I can’t walk round to the shop all the way and then come back. I could go one way, but I couldn’t come back. Sarah, 49

Interviewees reported changes to their ability to undertaking shopping, engage in the work that they were previously employed in, as well as changes to hobbies or sports. Most striking were references to resultant inability by some to engage in social or family activities.

I don’t go out. I don’t walk as much. I don’t participate in going out with family and the like as much as I used to. Greg, 65

One interviewee spoke of stopping routine running he had engaged in for a number of years, firstly due to uncertainty about whether he could run following diagnosis, although he received no advice in relation to this, and subsequently due to worsening levels of fatigue.

I was running six miles a week, every week, and I’ve been doing that for three years, so it’s come as a big hell of a shock. I can’t run anymore … I didn’t want to pack up, I loved it, definitely. Because I’d run with my daughter, so father and daughter, we loved it. It was really bonding and good. Dave, 68

What was clear on questioning most participants about the changes to their levels of activity or reduced ability to function was that it clearly had an impact of their perceived QOL, but nearly all had not brought up these changes with their healthcare team or received advice on how to manage the effects of treatment on daily life. Twelve of the eighteen participants interviewed could not recall receiving any advice regarding PA since diagnosis. Others mentioned being aware of references to keeping active in leaflets they were given at the beginning of treatment.

#### Encouragement to be physically active and specific advice would be welcomed

Given that the majority of interviewees could not recall ever discussing PA as part of their treatment, the qualitative interview was the first discussion on this topic for most. Universally across the dataset there was a sense of agreement that PA and exercise should be encouraged by the healthcare team. Many expressed a desire to receive encouragement and reassurance about continuing to be active.

It was the first time I’ve actually heard anybody talking about supporting myeloma patients with exercise, and that was exactly what I had been looking for, to support my recovery, much earlier on. Lara, 66

Some expressed a preference to receive specific instructions regarding how much activity to do or to receive a prescribed program and that having to report back to a professional might facilitate participation. For one interviewee welcoming PA advice, simply receiving information on the exercise research study prompted him to be active.

More formalising the encouragement to do exercise, because it’s easy not to do exercise and just sit on a chair and fall asleep again as opposed to getting up and actually sort of doing something, which as I said, the paperwork did inspire me to do some. Greg, 65

Most interviewees were generally supportive of the exercise research study as a good idea, despite not suiting them to take part. A number of them enquired during the interview about specific components of the planned exercise study and whether they were things that they could undertake independently. This demonstrated a desire among patients to seek further information regarding exercising at this point in their treatment journey.

#### Vague, over cautious or lack of response to requests for advice

Some interviewees shared experiences of seeking or receiving advice around PA or functional concerns. Two participants who had spinal disease related to their myeloma and had been required to wear a hard, spinal brace as part of their treatment shared similar experiences around lack of advice living in and out of their brace. Both recalled receiving basic information at the fitting of their brace but felt that the little follow-up advice or practical review from physiotherapy was not in keeping with what they expected to happen during this process.

[It was] excellent in terms of getting the brace fitted and applied, but when I went to have the brace removed, it was literally a case of, “off you go,” and there was no real physio given at that stage, so it must be [their] opinion that no physio is required … I felt personally that was perhaps lacking in the treatment, or perhaps it wasn’t explained to me why no physio was required? Pete, 58

There was a sense from many that clearer responses to requests for information on PA would be welcomed, for some any response at all would have potentially helped them continue to keep active. A perception of being “left to it” featured among the interviews with regard to physical consequences of treatment up to this point in their journey to ASCT.

We did ask various questions about, “Was she familiar with a series of exercises I could do at home?” and, basically, I haven’t had any response from that … I only just get asked basic questions about my wellbeing and the only thing was, “Are you getting out and about? Are you able to walk?” and that was basically it, end of conversation regards that … It’s almost as if you’ve got the medical treatment and then you’re basically just left to your own devices to sort of like recover in your best way possible physically. Andy, 73

One example described by an interviewee, was their experience attending a generic pre-chemotherapy induction information session with their partner. It provided a stark insight into how delivery of advice from health professionals can have profound consequences on how someone may adapt their life to such advice. Over-cautious advice surrounding avoiding scenarios where the patient went out alone, instilled a fear in both them and their partner that resulted in restrictions of activity outside of the home, made worse by the fact their partner worked during the day.

When I went first to my chemotherapy, they had a nurse give you an introduction or an induction course, and they said to me, well they said to all of us “Don’t go out exercising or walking unless you have someone with you.” That goes in the back of your mind and you think I’d better not go out. You know. That’s quite a downer to be honest … It’s just the fact that they said “Don’t go walking unless someone’s with you” and you think hello, well you’d better not do anything then. That’s probably why I don’t go anywhere or do anything. Plus my partner was with me and she said to me “Don’t go out unless I’m with you” so she goes to work all day and I’m at home. So I don’t go out, which is criminal really I suppose, isn’t it? I should go out. Interviewer: How does it make you feel then having that information given to you? Very cautious, I just feel cautious, maybe I shouldn’t go out, I’d better not go out, and if someone doesn’t find me, I fall over or something … John, 72

The general feeling from the data was that input for advice and tailored support in relation to PA was lacking, most did not recall any conversations about PA or physical recovery and there were explicit references to lack of access to physiotherapy.

### Common treatment side-effects are expected and endured but personal impact underestimated and unaddressed

Exploring experiences of discussing exercise and PA during treatment led to interviewees highlighting the impact of treatment side-effects they had experienced and naturally resulted in discussion surrounding advice or support given for managing these consequences of treatment. As previously described, fatigue was the most commonly reported, but weakness, breathlessness, poor appetite, gastrointestinal symptoms, “chemo brain,” as well as the role of steroid medication impacting upon sleep and weight were all mentioned.

#### Side-effects are considered inevitable but individual impacts are wide ranging

Interviewees shared a level of awareness of these symptoms as side-effects associated with myeloma and its treatment through the information received at the beginning of treatment, mostly in packs of written resources. There was no expression of surprise or unawareness that these side-effects would occur, but a level of inevitability did appear to result in lack of action in reporting these concerns, and particularly in discussing the impact on aspects of their everyday lives, with their medical teams. For some there was a sense of expecting more input from their health professionals but also an acknowledgment that they themselves did not initiate seeking additional input for side-effects and therefore it may be their own fault they did not have any.

I think it’s probably in a sense my own fault, because if I had attended the classes and the clinics I might have found out a few of these things because other people are there. But I’m not one for pushing that forwards. [CNS] said “I’ll look after it,” didn’t she? Her words were, before I started the treatment, “Don’t worry, I’ll look after you” and then I never saw her again for four months … She did reassure me and then you’re left on your own. I’m not saying I’m strong-willed or minded but I can see someone with less than I’ve got who would really take it to heart and really worry about it, rather than try and sort it out yourself, or put up with it or make allowances for it or try and get through it how you would get through it, without any advice from her. Dave, 68

A number of participants stressed the concept of “getting through it” and looking forward to completing their induction chemotherapy soon with a presumption that side-effects would subside, therefore did not see any point in raising concerns.

*They tick it off on a chart and that’s about it really. They don’t do anything. I can’t see what else there is to do. You’ve got to have the treatment, so it’s one of the side effects. Can’t really do anything about it I suppose … I’ve only got one cycle left anyway so it doesn’t matter*. Sally, 41

As I say, the attitude is just, ‘Sadly that’s part of the effects of the treatment. Jane, 73

Although some interviewees shared their insights into the possibility that their upcoming ASCT may worsen or lead to new side-effects, many did not acknowledge that this next phase of treatment could possibly lead to or sustain consequences of treatment beyond the end of their induction chemotherapy.

#### ‘You’re getting away lightly’ (minimization of side-effects/concerns)

Central to the discussions surrounding reporting of treatment side-effects was the notion that their experiences are normal or par for the course; others reported they were made to feel that their experience may not be as bad as it could be or it could be worse, reinforcing the tendency to underreport.

I suppose it’s the medication I have. It does make me very tired, which gets mentioned a lot when I go for the pre-chemo treatment, but again I’m always told that is normal, and that is part of it. Jane, 73

Across the dataset there was sharing of incidences recalling when asked by their healthcare team about side-effects, interviewees projected a feeling that this was generally “tick box” questioning with little potential to discuss the individual impact of side-effects on their life. There was also a perception that their concerns were minimized to not being as bad as others or as they could be. Some questioned that despite being told their negative consequences of treatment were not “too bad” comparatively and understanding that their myeloma was improving, why they did not feel better or when would they feel better?

They did say “Your side-effects etcetera, you’re doing well, you’re getting away with … ” And every time I have the testing, “Oh yes it’s good, it’s improving, it’s better.” I say “Well why don’t I feel better?” And they said “Well, it will come.” Long time coming … Dave, 68

There was an impression across the data that perhaps interviewees felt their concerns were not considered comprehensively enough up to this point in their treatment. Although interviewees shared experiences of lack of enquiry or minimization of their concerns by their medical team, it was also clear that interviewees did not initiate reporting of some of their concerns, particularly where symptoms impacted on daily activities, to their healthcare team.

## Discussion

This qualitative interview study explored the experiences of people with myeloma, recently referred to a specialist cancer center for consideration for ASCT, who had been approached for and declined to take part in a pilot RCT of an exercise intervention delivered pre- and post-ASCT. The research questions focused on investigating reasons for declining the exercise RCT and experiences of discussing PA with their healthcare team and the themes that were shaped from the dataset sit under the following broad areas: engagement with and interpretation of information related to their disease, its treatment and the research study; paucity of advice regarding PA despite an awareness of its benefit and desire to receive it; and deficiencies in communication related to side-effects and concerns.

As expected distance and travel requirements to reach the specialist center was the overwhelming reason for declining to take part in the exercise RCT. Travel or distance is a common reason cited by many cancer exercise RCTs when reporting uptake to trials (Sheill et al., 2019), but there is little literature exploring in depth, the reasons people do not participate in studies of this type. This qualitative study discovered deeper understanding of this common reason for declining trials. Underlying the logistical challenges of traveling or distance were concerns related to fitness, mobility, levels of fatigue, confidence traveling alone as well as the existing time burden of attending specialist care at a tertiary cancer center.

One qualitative study exploring reasons for declining a community-based walking program in primary care asked those declining the trial to complete a non-participant questionnaire and conducted qualitative interviews in a purposeful sample of 30 decliners. Reasons for not enrolling in the RCT were categorized into internal (i.e. medical problems and perception of being fit enough) and external motives (i.e. travel required and commitments) (Normansell et al., 2016). Interpretation of reasons for declining our pilot RCT indicates that participants’ internal and external reasons were interconnected. Travel as an external factor was very much more problematic for many due to internal factors of perceived lack of fitness, mobility and symptom concerns. The interconnectedness of these issues is of importance as participants felt that the exercise intervention would benefit them in terms of potentially improving their fitness. Most recognized that their fitness or functional capacity limited their ability to travel more regularly or utilize public transport instead of more costly hospital or private hire transport.

Other important experiences related to travel as a given reason for declining included the cumulative time burden of travel and time spent waiting in clinic. The design of the original face-to-face study assessments to coincide with clinic visits was therefore not as convenient as researchers initially believed, and some participants were put off by the perception that attending the intervention would result in a “whole day” time burden similar to that experienced with a clinic visit. These findings resonate with quantitative data reported in a systematic review of exercise studies in advanced cancer which found lack of time, multiple hospital commitments and transport issues to be the most commonly cited reasons for declining participation (Sheill et al., 2019). Mawson et al. (2021) conducted a single-arm feasibility study of prehabilitation for myeloma patients awaiting ASCT and included qualitative interviews of study decliners. Although only briefly described alongside quantitative study results, their qualitative findings of six non-participants are very closely aligned with the reasons for declining reported in this study. They report that although distance to travel and the location of the exercise venue were the most common reasons given by non-participants, the impact of fatigue, reduced ability to engage in activities of daily living (ADLs) and management of clinical appointments were also highlighted as important reasons for declining (Mawson et al., 2021).

Myeloma survivors stress a desire for individualized exercise support programs delivered by professionals with knowledge of the disease (Craike et al., 2017; Land et al., 2022). Physiotherapists are seen by patients to be best placed to provide exercise support (Craike et al., 2017; Nicol et al., 2020) with supervised sessions motivating patients to adhere to exercise prescriptions and challenges maintaining similar levels of exercise in unsupervised sessions (Land et al., 2022). However, preference for location of such exercise programs is mixed. Some studies have found equal preference for programs based at specialist myeloma center and for home-based programs (Craike et al., 2017). More recent survey-based research among Australian myeloma survivors found preferences for programs that were flexible in terms of time and delivered close to home, with no clear preferences related to specific location in terms of clinical centers or within home (Nicol et al., 2020). Increased experience of remotely engaging in virtual health delivery through different forms of telehealth offers an opportunity to explore the effectiveness of remotely supervised, home-based exercise interventions (Bland et al., 2020). A virtual approach that incorporates the specialist knowledge and supervision by physiotherapists delivered conveniently at home would eliminate the barrier to participation related to travel and may appeal to the preferences of exercise delivery.

In contrast to other literature that has found perceptions of already being active enough or not being interested in PA as significant reasons for declining recruitment to PA studies (Attwood et al., 2016), interviewees shared an understanding of being physically active as important. Positive beliefs related to exercise and its role in managing symptoms and recovery from cancer treatment among myeloma survivors have previously been reported (Coon and Coleman, 2004; Craike et al., 2013, 2017). Indeed, more physically active myeloma patients have less comorbidity, improved tolerance of treatment and potentially respond better to treatment (Moller et al., 2021). Interviewees were welcoming of PA advice but most reported not receiving any since diagnosis. This is in line with research exploring provision of PA advice among those with cancer (Barnes and Schoenborn, 2012; Fisher, Williams, Beeken, and Wardle, 2015) and specifically myeloma (Nicol et al., 2020; Walpole, Clark, and Dowling, 2018). Lack of specialist or reliably informed advice may also result in missed opportunities to receive trustworthy, encouraging advice about being physically active especially among those participants who experienced receiving vague or over-cautious advice to avoid activity during their treatment. Although lack of discussion of PA advice was an unsurprising finding, the additional findings around underreporting or lack of communication of treatment side-effects and impacts on ADLs with their clinical teams were.

The concept of supportive care in cancer is considered a cornerstone to the management of the disease and is defined as “the prevention and management of the adverse effects of cancer and its treatment” (Rittenberg, Johnson, and Kuncio, 2010). Good supportive care includes education regarding consequences of treatment and routine screening for side-effects to provide opportunities to discuss their experience of cancer treatment and any effects on their QOL with their clinical team (Olver et al., 2020). Our interviewees were generally well informed regarding the consequences they expected from treatment in terms of side-effects but appeared to underestimate the effects on their day-to-day lives and ability to maintain ADLs, particularly social engagement. What was striking was the realization that they had often not informed their clinical teams of the effect that treatments and living with myeloma were having on their ability to engage in usual activities. More often they shared a feeling of not being asked or that enquiry was tokenistic in nature, therefore, held assumptions that nothing could be done to mitigate. It is also important to highlight that interviewees were all ASCT eligible patients considered the “most fit” of myeloma patients undergoing treatment and mostly only a few cycles into their induction chemotherapy but yet their PA and daily lives were already hugely impacted by treatment so far, and so concerns are likely to be much more evident in those having subsequent lines of treatment following relapse.

Interviewees were wholly accepting of the individualized approach taken to recruit for the exercise RCT. The appeal and resounding positive experiences related to receiving an individualized approach for the lifestyle-related research trial was clear from most interviewees. The addition of the interview drew out concerns related to their treatment to date and how it related to inactivity and changes to everyday living. The approach by telephone followed by face-to-face encounter with researcher and written information resources provided in between provided them with adequate information to make a personal decision to take part. Participants reported variable engagement with the written study information. Although written study information is required and known to benefit research participants by allowing time for reflection, trial information delivered verbally, in person by an approachable clinician or researcher with good communication skills is known to be preferred by those being approached for RCTs (Houghton et al., 2020).

Given the acceptability and positive experiences reported by interviewees of being sought out to receive a one-to-one interaction and follow-up discussion related to research participation, could this model of approach be incorporated into clinical services to enhance individualized supportive care? Participants shared a recognized gap in provision of opportunity to comprehensively discuss their disease and ongoing treatment and expressed a desire to receive guidance and support for PA, which may extend to other lifestyle and self-management interventions. The definition of supportive care includes reference to enhancing rehabilitation and survivorship as integral to supportive care (Rittenberg, Johnson, and Kuncio, 2010), yet despite substantial evidence base for inclusion of a rehabilitative approach to address symptoms as part of clinical pathways, the provision of such is sparse and subject to much variability (Robb and Davis, 2015; Transforming Cancer Services Team, 2019). Approaching provision of PA/lifestyle advice or review of symptoms and concerns by using a process similar to research trial recruitment may be valuable to patients, but rehabilitation goes far beyond just delivery of exercise (Silver et al., 2015). Delivery of a model of supportive care that encompasses all its intended elements requires coordinated, multidisciplinary collaboration built around patients and accessible across the continuum of cancer treatment (Bayly and Lloyd-Williams, 2016; Olver et al., 2020; Silver et al., 2015; Snowden et al., 2017). Exploring with cancer survivors how well equipped they are to manage their recovery as well as provision of generic information regarding life after treatment could be delivered within patient education programs (Walpole, Clark, and Dowling, 2018). However, offering individualized opportunities to discuss consequences of treatment specific to them and their impact on their life, with relevant resources and time to consider and reflect before a follow-up discussion, may provide better conditions to deliver holistic, biopsychosocial approach to care and instigation of signposting for psychological support and early rehabilitation to minimize symptoms and decelerate or prevent impact on function.

This study is not without its limitations. Like all qualitative research, the role of the researcher will have influenced all elements of this study. The lead author was chief investigator and sole recruiter for the pilot exercise RCT for which these interviewees were approached for and this may have led to mostly positive feedback regarding research approach. This known “ownership” of the study may have led interviewees to consider their responses differently than if approached for a generic study recruited to by a research team.

Like much exercise and lifestyle-related research, the pilot RCT and subsequent qualitative decliner study may also have attracted participants already interested in being physically active or seeking support for PA. The participants were purposefully recruited from one center, and this limits the transferability of these findings to other myeloma survivors and those in other locations. However, it is positive to note that there was a good response from decliners to taking part in this interview study (83%) and that the findings of this study do align with other qualitative literature.

Finally, due to this study being conducted and completed prior to the COVID-19 pandemic, discussion about virtual delivery was not part of this interview but may have revealed useful insights into differences in engagement with research recruitment and communication of consequences of treatment that do not feature in this dataset.

## Supplementary Material

Supplemental Material
