# Supplementary material for: The views and experiences of people with myeloma referred for autologous stem cell transplantation, who declined to participate in a physiotherapist-led exercise trial: a qualitative study
Source: Physiother Theory Pract. 2023 Aug 9;40(10):2331–43. doi: 10.1080/09593985.2023.2244068 (PMC11458126; doi:10.1080/09593985.2023.2244068)
Supplement: Supplemental Material [file IPTP_A_2244068_SM9611.pdf]

Myeloma Transplant Exercise Study
Interview Schedule v2 – People who decline to take part in trial

This guide is to ensure key aspects are covered during the interview. However a respondent-sensitive approach should be taken, allowing deviation from question order and raising additional issues if desired.

Interviewers should be mindful of the sensitive subject of the interview.

Introduction
Introduce yourself and explain purpose of the interview: to explore the participant's experience of being approached to be a participant in the trial and the reasons for deciding not to take part. Ask permission to record the conversation. Assure confidentiality and ask participant to be as open and honest as they can be.

1. I understand some time has passed since you were sent the letter about the research study. If I can ask you to think about the study, do you remember <u>what it involved</u>?
    - Can you recall what the study assessments involved?
    - What was the study intervention?
    - Can you recall anything about allocation of a group…?

2. Can you give some detail [for the recording] about <u>your reason for deciding not to take part</u> in this study?
    - *Probe:* If distance/journey is given as reason – asking them to explain their journey to UCLH, what does it involve, how long does it take etc
    - If symptoms/side effects of myeloma/treatment given – explore how they experience these symptoms, how they make travelling/doing things/life difficult, how do these changes make them feel?
    - Have you sought advice from anyone regarding these symptoms/changes to activities

3. If you can think back, what do you remember about your <u>experience of being approached</u> about the study and being asked to take part?
    - *Probe:* Do you recall who approached you? What was their manner? Did you receive adequate information?

We would like to know more about the process people go through when they are asked to take part in a study like this one. I am going to ask a few questions and I would like to know your thoughts or feeling about these different parts of the process of being asked to take part in this research study.

4. Did you read the information leaflet in full?
    - *Probe:* What did you think about the length of it? About the language? Was it easy to read/follow/understand?
    - If not, what put you off reading it in full?

5. What did the information in the leaflet make you think about?

6. Did you weigh up any positives and negatives about taking part?
    - *Probe:* What were the negatives/positives for you?
    - Person may already have given negatives in earlier questions, so perhaps explore positives if not yet discussed

7. Did you discuss it with anyone else?
    - *Probe:* If so, with whom? What did they say? Did their opinions/thoughts affect your decision or thinking?

8. Do you have any experience of taking part in research before?
    - If yes, PROBE

9. How did you feel about being contacted about the study by the research team rather than by the consultant or clinical nurse specialist?
    - Would being sent information about this study through your clinical team changed your decision?

10. Have you received any information about physical activity since your diagnosis?
    - *Prompt:* If no, would they have liked advice during treatment
    - If mentions previous activity now reduced, explore why they do less – were they advised to reduce/stop their activity/exercise or did they arrive at the decision to stop themselves and why?

11. Has being approached about this research study changed your thoughts or opinions on physical activity or exercise?
    - *Prompt*: will you do anything differently in preparation for your transplant?

12. Is there anything else you would like to say about the trial, that we've not already talked about?

Concluding comments and thanks
Thank the participant for their time today and ongoing and let them know where they can contact you in the future if they do have any additional comments.
